# Causal association between circulating inflammatory proteins and peripheral artery disease: a bidirectional two-sample Mendelian randomization study

**DOI:** 10.3389/fimmu.2024.1432041

**Published:** 2024-08-16

**Authors:** Juncheng Zhao, Bo Sun, Shujie Huang, Yunhui Chen, Jingqiang Yan

**Affiliations:** Department of Vascular Surgery, Qingdao Municipal Hospital, Qingdao, China

**Keywords:** peripheral artery disease, circulating inflammatory proteins, genome-wide association study, Mendelian randomization, causal association

## Abstract

**Introduction:**

A growing body of research has shown a strong connection between circulating inflammatory proteins and Peripheral artery disease (PAD). However, the causal relationship between circulating inflammatory proteins and PAD is still not fully understood. To investigate this association, we conducted a bidirectional Mendelian randomization study.

**Materials and methods:**

Our study utilized genetic variation data obtained from genome-wide association studies (GWAS) datasets. Specifically, the GWAS dataset related to PAD (identifier: finn-b-I9_PAD) included 7,098 cases and 206,541 controls. Additionally, we extracted data on 91 inflammatory proteins from another GWAS dataset (identifiers: GCST90274758-GCST90274848), involving 14,824 participants. To assess the causal relationship between circulating inflammatory proteins and PAD development, we employed methodologies such as inverse variance weighting (IVW), MR Egger regression, and the weighted median approach. Furthermore, sensitivity analyses were conducted to ensure the reliability and robustness of our findings.

**Results:**

Two inflammatory proteins were found to be significantly associated with PAD risk: Natural killer cell receptor 2B4 levels (OR, 1.219; 95% CI,1.019~1.457; P=0.03), Fractalkine levels (OR, 0.755; 95% CI=0.591~0.965; P=0.025). PAD had statistically significant effects on 12 inflammatory proteins: C-C motif chemokine 19 levels (OR, 0.714; 95% CI, 0.585 to 0.872; P=0.001), T-cell surface glycoprotein CD5 levels (OR, 0.818; 95% CI, 0.713 to 0.938; P=0.004), CUB domain-containing protein 1 levels (OR, 0.889; 95% CI, 0.809 to 0.977; P=0.015), Fibroblast growth factor 23 levels (OR, 1.129; 95% CI, 1.009 to 1.264; P=0.034), Interferon gamma levels (OR, 1.124; 95% CI, (1.011 to 1.250); P=0.031),Interleukin-15 receptor subunit alpha levels (OR, 1.183; 95% CI,(1.005 to 1.392); P=0.044), Interleukin-17C levels (OR,1.186; 95% CI, (1.048 to 1.342); P=0.007), Interleukin-1-alpha levels (OR, 1.349; 95% CI, (1.032 to 1.765); P=0.029), Interleukin-5 levels (OR, 1.119; 95% CI,(1.003 to 1.248); P=0.043), Latency-associated peptide transforming growth factor beta 1 levels (OR,1.123; 95% CI, (1.020 to 1.236); P=0.018), Matrix metalloproteinase-10 levels (OR, 1.119; 95% CI,(1.015 to 1.233); P=0.024), Signaling lymphocytic activation molecule levels (OR, 0.823; 95% CI, (0.693 to 0.978); P=0.027).

**Conclusion:**

Our research expands on genetic studies exploring the strong association between circulating inflammatory proteins and PAD. This discovery has the potential to inform and shape future clinical and basic research endeavors in this area.

## Introduction

Peripheral artery disease (PAD) refers to the narrowing or blockage of arteries due to the accumulation of fatty deposits and atherosclerosis (1). It is a major contributor to heart disease worldwide, resulting in significant morbidity and mortality (2). PAD significantly indicates both overall mortality and cardiovascular-related mortality. Research has demonstrated that PAD increases the risk of overall mortality by 60%, cardiovascular-related mortality by 96%, coronary artery disease by 45%, and cerebrovascular disease by 35% (3). Atherosclerosis is a chronic inflammatory disease with autoimmune components, attracting cells from both the innate and adaptive immune systems into atherosclerotic plaques (4, 5). Macrophages, along with vascular smooth muscle cells, produce various pro-inflammatory mediators, further amplifying the inflammatory response (6). Zhao et al. extended their previous work by conducting a genome-wide protein quantitative trait loci (pQTL) study, which identified the genetic architecture of 91 circulating inflammatory factors in a cohort of 14,824 participants of European ancestry (7). Multiple recent studies have incorporated these 91 inflammatory proteins into MR studies, thereby broadening the scope and application of inflammatory proteins in MR studies even further (8, 9). A few circulating inflammatory proteins have been reported to be closely correlated with PAD in previous studies—for example, TNF-α, IL-8, MMP-2 and MMP-9 (10, 11). The circulating inflammatory proteins may provide valuable predictive information for improving the prognosis of PAD, which is also one of the enormous potential strategies for PAD prevention and treatment. However, observational studies can be confounded by potential biases and reverse causality. The relationship between inflammatory proteins and PAD observed in previous observational studies requires further investigation.

Mendelian randomization (MR) is a robust method used in epidemiological research to assess causal relationships between risk factors and specific diseases by utilizing genetic variations as instrumental variables (IV). By leveraging genetic diversity, MR approaches aim to disentangle causal associations between exposures and outcomes, effectively addressing inherent confounding factors in epidemiological research. Through the application of MR methods, genetic variability adheres to the principle of random allele assignment during meiosis, similar to the randomization process in controlled trials. This distinctive feature empowers MR to overcome challenges such as confounding biases, reverse causation, and biased sampling commonly encountered in observational studies, while circumventing the practical constraints of randomized trials. Despite its versatility, MR has not yet been explored as a tool for investigating potential causal relationships between PAD and circulating inflammatory proteins. To bridge this gap, we conducted a bidirectional MR study to elucidate potential associations between PAD and circulating inflammatory proteins

## Materials and methods

### Study design

**Figure 1:**
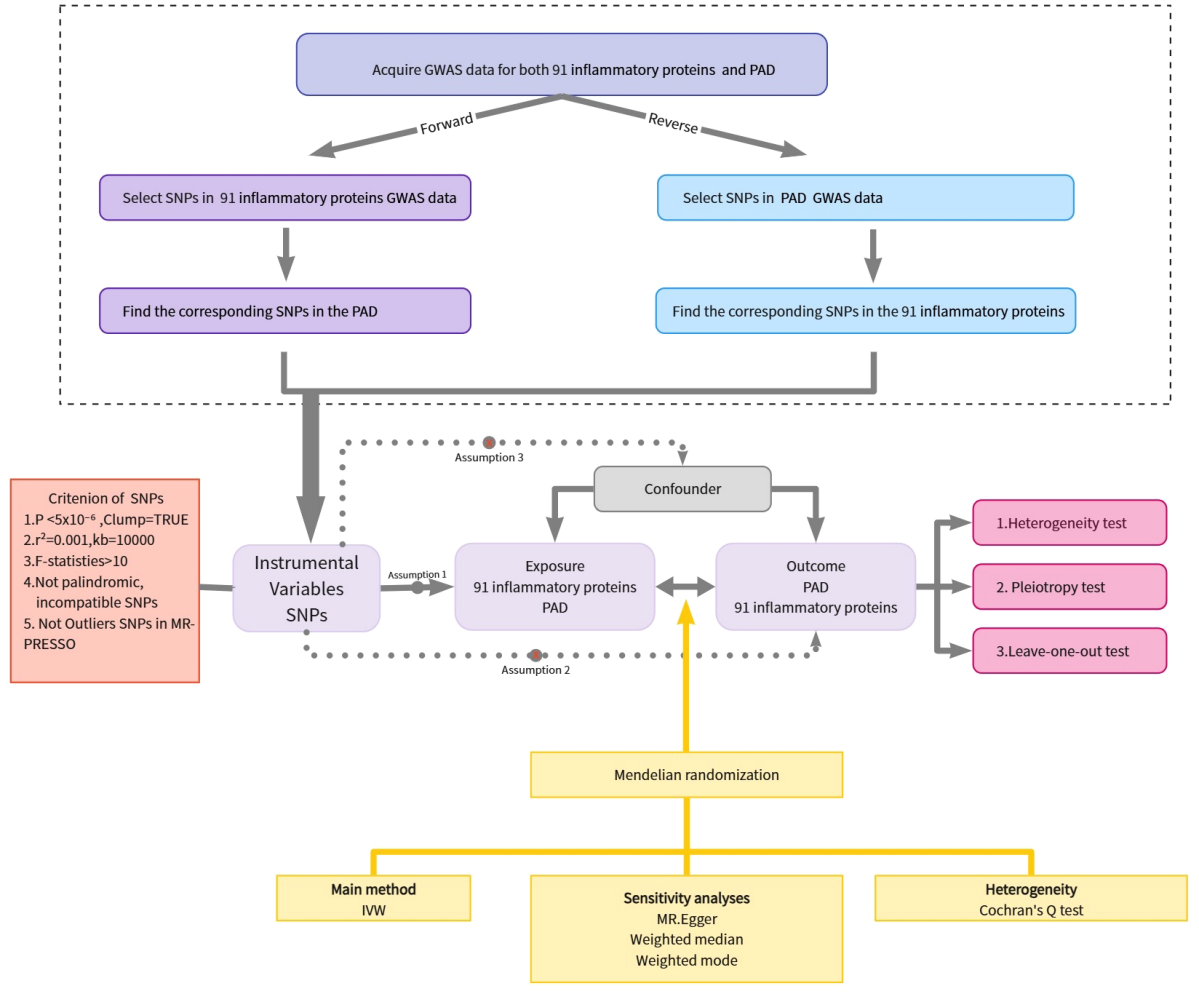
Overview of the assumptions of the Mendelian randomization design and the study design. GWAS, genome-wide association study; IVs, instrumental variables; PAD, Peripheral Artery Disease; SNPs, single-nucleotide polymorphisms.

The study employed a two-sample MR analysis to investigate the potential causal associations between 91 inflammatory proteins and PAD. MR analysis is a methodology that seeks to uncover causal associations between exposures and outcomes by using genetic variants as proxies for exposure. It should be emphasized that the IVs used in MR analysis must meet three crucial prerequisites: (1) genetic variants should demonstrate a robust association with the environmental exposure factors, (2) genetic variants should be independent of potential confounding factors, and (3) the genetic variants should solely affect the outcome through the exposure route (12, 13). **Figure 1** depicts the flowchart of the MR study process.

### Ethics statement

The study sample consisted of human individuals and involved a secondary analysis of preexisting data, obviating the need for ethical approval.

### Data source

The genetic data used in this analysis were obtained from publicly accessible GWAS and UK Biobank summary datasets. These datasets are readily available to anyone and do not raise concerns about conflicting interests. The information on the research population is from the Finnish dataset. Specifically, data on PAD includes 7098 cases and 206541 European controls. Furthermore, data on 91 inflammatory proteins were collected from a research project investigating associations with genetic variations (7). The research included eleven groups, involving a combined total of 14,824 individuals. Genetic data spanning the genome and plasma protein information were collected using the Olink Targeted Inflammation Panel, with all participants providing prior written consent.

### IV selection

After gaining a comprehensive understanding of MR analysis, the criteria for selecting IVs are as follows:1.Strong correlation with exposure: The IV should demonstrate a strong association with the exposure factor, meeting a significance threshold of p < 5e-8 and an f-statistic exceeding 10.2.Confounder independence: The selected IVs must not be influenced by potential confounders that could bias the relationship between exposure and outcome.3.Unidirectional influence: The IV should only affect outcomes through exposure variables and should not directly influence outcomes themselves. Adhering to these principles ensures logical consistency in causal analysis.

Linkage disequilibrium (LD) is characterized by the non-random association of specific alleles within a chromosome, often attributed to the close proximity of genes in the genome. To maintain the randomness of genetic variation, a core assumption of MR it is crucial to minimize LD between IVs in analysis. To maintain research integrity, criteria were established to set thresholds for two parameters, r2 and kb. The threshold for r2 was set to be less than 0.001, while for kb, it was set to be less than 10,000. These criteria allowed excluding single nucleotide polymorphisms (SNPs) within a 10,000 kb range that had an r2 value exceeding 0.001 compared to the most significant SNPs. By rigorously applying these selection criteria for IVs, our research aims to maintain the validity and credibility of our causal inference analyses.

### MR analysis

The MR analysis utilized the ‘Two Sample MR’ package in R version 4.3.1, employing three distinct statistical techniques: inverse variance weighting (IVW), weighted median estimation, and MR-Egger regression. Initially, an individual Wald ratio was computed for each SNP to assess its association. Subsequently, the IVW method combined these ratios using IVW to assess the relationship between 91 inflammatory proteins and PAD. Recognizing the limitations of the IVW method is crucial, as it presupposes that all genetic variations serve as valid IVs. However, this assumption may not always align with practical contexts, requiring careful consideration (14). Acknowledging the potential limitations of this presumption, alternative statistical techniques have been incorporated. Significantly, this tactic can produce reliable assessments when more than half of the weighting corresponds to credible IVs (15). The MR-Egger regression method was also implemented. This approach provides a valuable improvement by relaxing the assumption that all genetic variants act as valid IVs, thereby offering a useful enhancement. Our use of the Inverse Variance Weighted (IVW) method, MR-Egger regression, and the Weighted Median approach provides a comprehensive analysis of the genetic variants as instrumental factors. These methods collectively strengthen our ability to estimate stable cause-and-effect relationships, even when individual genetic variants may not qualify as strong instruments on their own. The false discovery rates (FDR) correction is used to control the number of false positive events in multiple comparisons by correcting the *P*. The corrected *P* is called *P _FDR* and the *P _FDR* < 0.2 is considered statistically significant. FDR thresholds can be adjusted to suit specific research contexts and needs. In some cases, it is reasonable and beneficial to set higher FDR thresholds (e.g., 0.15, 0.2) (16, 17). Using a higher FDR threshold (e.g., 0.2) can increase the likelihood of finding significant results in a given context while still maintaining reasonable error control. Therefore, in this study, a higher FDR threshold was set to better detect the risk factors for PAD, thus providing new insights for the prevention and treatment of PAD.

### Sensitivity analysis

The study utilized version 4.3.1 of R for all analyses. Using the R packages ‘Two Sample MR’ and ‘MR-PRESSO’, MR analysis and data visualization were conducted. Various methods, including IVW, MR-Egger, WM, and weighted mode, were employed to investigate the causal association between 91 inflammatory proteins and PAD. MR-PRESSO analysis was utilized to assess the bidirectional causal association between these proteins and PAD. The primary method in our MR research was IVW analysis, which excludes intercept terms in regression and relies on outcome variance for reliable estimates, particularly in the absence of directional pleiotropy in IVs (18). To assess the diversity of the chosen independent variables, Cochran’s Q statistic and the associated P-values were utilized. Upon rejection of the null hypothesis, the random effects IVW method was chosen over the fixed effects IVW method (19). MR-Egger serves as an informative approach in MR, amalgamating information to assess result accuracy (20). In MR studies involving multiple genetic variants, the MR-Egger and WM methods serve as crucial sensitivity analyses. The MR-PRESSO technique was used to identify horizontal pleiotropy outliers in MR tests with multiple instruments at the summary level and to reassess causal effects after eliminating pleiotropic IVs (21). The scatter diagram suggests that outliers do not have a significant impact on the findings, while the funnel plot illustrates the robustness of the relationship in the absence of variability.

## Results

### Detail information of the included SNPs

Information regarding the MR investigation of PAD inflammatory proteins is provided in **Supplementary Table 1**. The IVW method employed a significance threshold below 0.05, and the MR examination presents all positive outcomes along with details about the genetic variants utilized, as stated in **Supplementary Tables 2**, **3**. This comprehensive collection includes various details such as genetic locus, effective allele (EA), and effective allele frequency (EAF). Notably, the f-value of the selected IVs exceeds 10. The F statistic serves as a crucial indicator of an instrument’s efficacy in MR analysis, with values exceeding 10 generally recognized as indicative of a reliable instrument (22, 23). Through systematic selection of SNPs, consistent associations with target exposures are ensured. Furthermore, efforts are made to effectively manage the relationships between outcome variables and potential confounders within acceptable parameters. Consequently, all selected SNPs can be considered dependable and versatile IVs.

### Exploring the causal links between inflammatory proteins and PAD through MR

**Figure 2:**
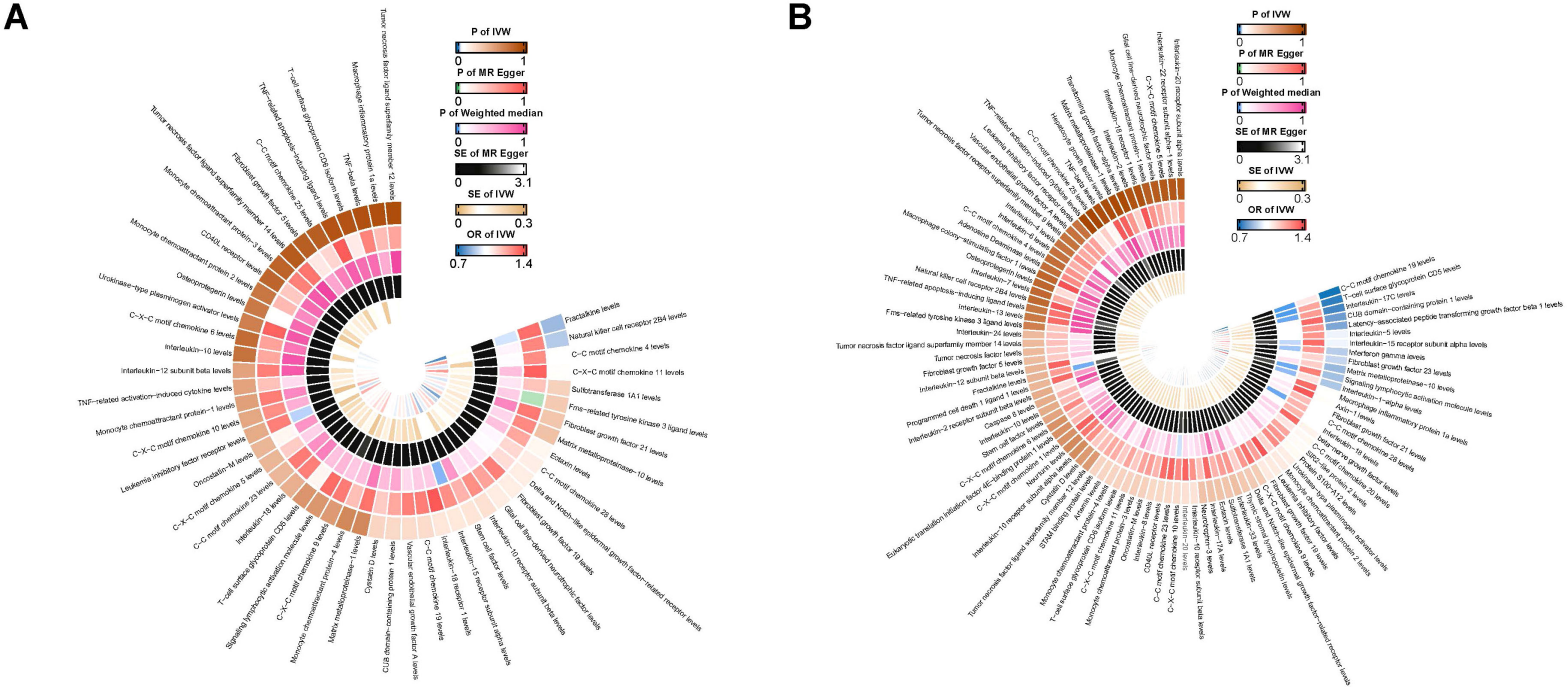
**(A)** Ring heat map of MR analysis of the causal association of circulating inflammatory proteins on PAD. **(B)** Ring heat map of MR analysis of the causal association of PAD on circulating inflammatory proteins. MR, Mendelian randomization; PAD, Peripheral Artery Disease.

**Figure 3:**
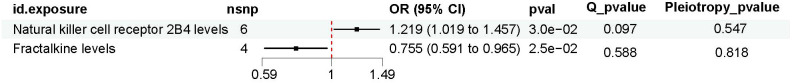
The causal impact of inflammatory proteins on peripheral artery disease.

A two-sample MR analysis was conducted to investigate the impact of inflammatory proteins on the causal relationship of PAD. In **Figures 2A**, **3**, the primary MR analyses of inflammatory proteins are displayed. Heterogeneity in the data was assessed using Cochran’s Q test, and if P exceeded 0.05, the IVW model with random effects was applied. The findings revealed a causal association between PAD and two inflammatory proteins: levels of Natural killer cell receptor 2B4 (OR, 1.219; 95% CI, 1.019-1.457; P=0.03) and Fractalkine (OR, 0.755; 95% CI, 0.591-0.965; P=0.025). In the absence of heterogeneity and horizontal pleiotropy, preference was given to the MR analysis over the IVW method. Furthermore, the lack of statistical significance in the discrepancy between the Egger intercept of MR-Egger and 0 (Pval > 0.05) suggested the absence of horizontal pleiotropy (**Table 1**). The results of the IVW, WM, and MR Egger mode for all circulating inflammatory proteins on PAD are displayed in **Supplementary Table 4**. **Supplementary Figures 1**–**3** illustrate the scatter plots, funnel plots, and leave-one-out analyses of inflammatory proteins on PAD.

**Table 1:** The heterogeneity and horizontal pleiotropy results of the 2 inflammatory factors and PAD in the forward MR analysis.

Exposure	Heterogeneity test						Pleiotropy test		
	MR Egger			IVW			MR Egger		
	Q-value	Q-df	p-value	Q-value	Q-df	p-value	Intercept	SE	p-value
Natural killer cell receptor 2B4	8.407	4	0.077	9.310	5	0.097	0.016	0.024	0.546
Fractalkine	1.854	2	0.395	1.923	3	0.588	-0.017	0.065	0.817

MR, Mendelian randomization; Q, heterogeneity statistic Q; df, degree of freedom; SE, standard error.

To further substantiate the findings of our Mendelian randomization analysis, we conducted validation using an independent dataset derived from the UK Biobank focusing specifically on PAD. This validation aimed to test the robustness and generalizability of our initial results, particularly concerning the impact of inflammatory proteins on PAD. The results are displayed in **Supplementary Table 5**. In the validation analysis, the findings revealed a causal association between PAD and five inflammatory proteins: Cystatin D levels (OR, 1.00069; 95% CI, 1.000-1.001; P=0.03), C-X-C motif chemokine 11 levels (OR, 1.002; 95% CI, 1.001-1.004; P=0.00004 < 0.001), Interleukin-8 levels (OR, 0.997; 95% CI, 0.995-0.999; P=0.032), Matrix metalloproteinase-10 levels (OR, 0.999; 95% CI, 0.998-0.999; P=0.032) and Oncostatin-M levels (OR, 1.002; 95% CI, 1.000-1.003; P=0.018).

### Exploring the causal links between PAD and inflammatory proteins through MR

**Figure 4:**
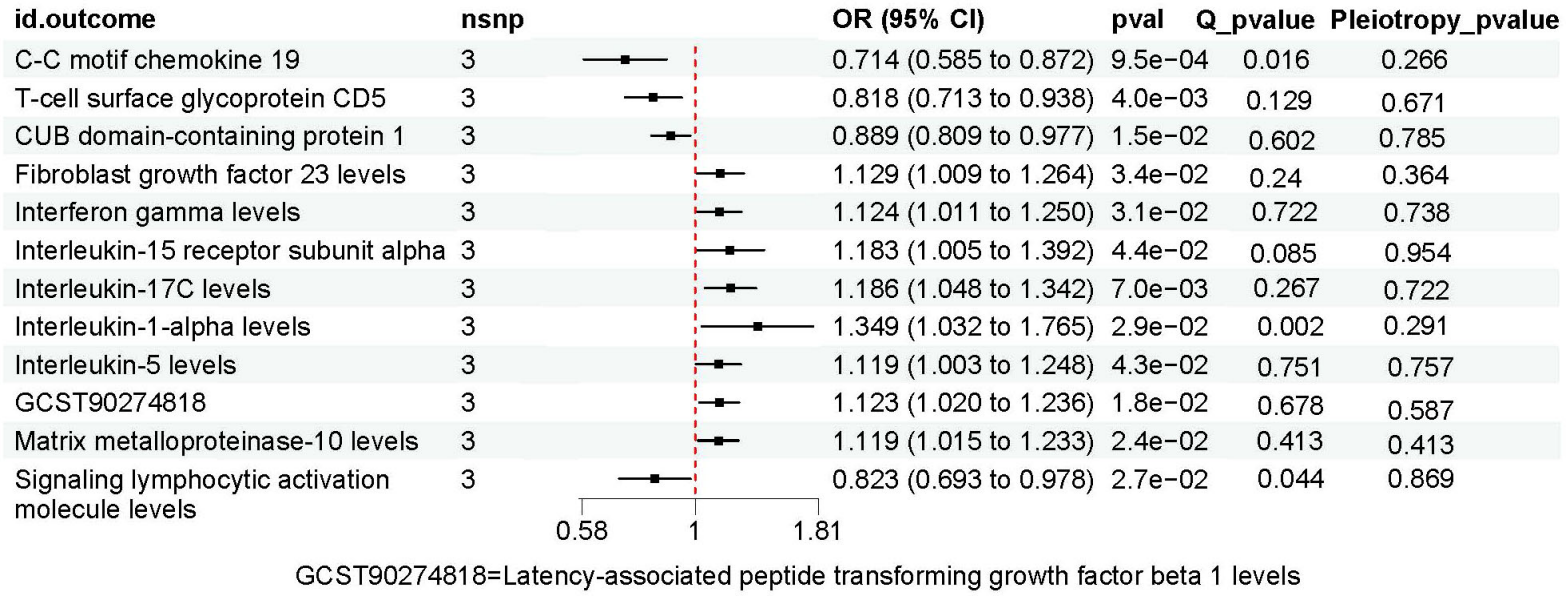
The causal impact of Peripheral Artery Disease on inflammatory proteins.

The results of MR analysis regarding the causal relationship between PAD and inflammatory proteins are shown in **Figures 2B**, **4**. Significant variations were observed in 12 inflammatory proteins (P < 0.05). Among these, PAD exhibited a protective effect for 4 inflammatory proteins: CC motif chemokine 19 levels (OR, 0.714; 95% CI, 0.585 to 0.872; P=0.001), T-cell glycoprotein CD5 levels (OR, 0.818; 95% CI, 0.713 to 0.938; P=0.004), CUB domain-containing protein 1 levels (OR, 0.889; 95% CI, 0.809 to 0.977; P=0.015), and Signaling lymphocytic activation molecule levels (OR, 0.823; 95% CI, 0.693 to 0.978; P=0.027). In contrast, PAD was associated with an increased risk for 8 inflammatory proteins: Fibroblast growth factor 23 levels (OR, 1.129; 95% CI, 1.009 to 1.264; P=0.034), Interferon gamma levels (OR, 1.124; 95% CI, 1.011 to 1.250; P=0.031), Interleukin-15 receptor subunit alpha levels (OR, 1.183; 95% CI, 1.005 to 1.392; P=0.044), Interleukin-17C levels (OR, 1.186; 95% CI, 1.048 to 1.342; P=0.007), Interleukin-1-alpha levels (OR, 1.349; 95% CI, 1.032 to 1.765; P=0.029), Interleukin-5 levels (OR, 1.119; 95% CI, 1.003 to 1.248; P=0.043), Latency-associated peptide transforming growth factor beta 1 levels (OR, 1.123; 95% CI, 1.020 to 1.236; P=0.018), and Matrix metalloproteinase-10 levels (OR, 1.119; 95% CI, 1.015 to 1.233; P=0.024). An analysis of data heterogeneity was conducted using Cochran’s Q test (P > 0.05), and a random-effects IVW model was employed. The Egger intercept of the MR-Egger analysis showed no significant deviation from 0 (Pval > 0.05), suggesting the absence of horizontal pleiotropy (**Table 2**). The results of the IVW, WM, and MR Egger mode for PAD on all circulating inflammatory proteins are displayed in **Supplementary Table 6**. Sensitivity analyses also confirmed the reliability of the causal relationships identified in the study. Scatter plots, funnel plots, and leave-one-out analyses of PAD on inflammatory proteins are shown in **Supplementary Figures 4**–**6**.

**Table 2:** The heterogeneity and horizontal pleiotropy results of the 12 inflammatory factors and PAD in the reverse MR analysis.

Exposure	Heterogeneity test						Pleiotropy test		
	MR Egger			IVW			MR Egger		
	Q-value	Q-df	p-value	Q-value	Q-df	p-value	Intercept	SE	p-value
C-C motif chemokine 19	1.357	1	0.244	8.269	2	0.016	0.249	0.110	0.265
T-cell surface glycoprotein CD5	3.100	1	0.078	4.099	2	0.128	0.092	0.163	0.671
CUB domain-containing protein 1	0.891	1	0.345	1.015	2	0.601	-0.032	0.091	0.784
Fibroblast growth factor 23	0.433	1	0.510	2.851	2	0.240	-0.141	0.091	0.363
Interferon gamma	0.461	1	0.497	0.651	2	0.721	-0.044	0.102	0.737
Interleukin-15 receptor subunit alpha	4.895	1	0.026	4.921	2	0.085	-0.016	0.222	0.953
Interleukin-17C	2.169	1	0.140	2.643	2	0.266	-0.071	0.153	0.721
Interleukin-1-alpha	2.426	1	0.119	12.438	2	0.001	-0.329	0.162	0.291
Interleukin-5	0.412	1	0.520	0.573	2	0.750	-0.042	0.105	0.757
Latency-associated peptide transforming growth factor beta 1	0.203	1	0.651	0.778	2	0.677	0.070	0.093	0.587
Matrix metalloproteinase-10	0.033	1	0.85	1.769	2	0.412	-0.124	0.094	0.4131
Signaling lymphocytic activation molecule	5.985	1	0.014	6.244	2	0.044	0.047	0.230	0.869

MR, Mendelian randomization; Q, heterogeneity statistic Q; df, degree of freedom; SE, standard error.

## Discussion

This research utilized a large dataset of publicly available genetic data to investigate the association between 91 inflammatory proteins and PAD. Initial findings in our study underscore the significant associations of certain inflammatory proteins with PAD risk. Notably, proteins like Natural Killer Cell Receptor 2B4 and Interleukin-17C have shown a substantial increase in PAD risk, highlighting their role as pro-inflammatory mediators. Conversely, Fractalkine emerges as a protective factor, suggesting a potential for therapeutic targeting.

The research suggests an association between elevated levels of the Natural Killer Cell Receptor 2B4 and an increased susceptibility to PAD. Interleukin-1-alpha, identified as a risk factor, may promote endothelial dysfunction and vascular inflammation, which are critical in atherosclerosis progression seen in PAD. On the protective side, Fractalkine could reduce the risk of PAD by inhibiting the recruitment and migration of inflammatory cells, thus diminishing vascular inflammation. CD244 (2B4) is present in memory phenotype CD8(+) T cells as well as in all natural killer cells. Unlike other inhibitory receptors, 2B4 remains unaffected by MHC class I molecules (24). Li et al. found that individuals with coronary artery disease show a higher frequency of spontaneous NK cell apoptosis (25). The Natural Killer Cell Receptor 2B4 is known for its ability to diminish the effector function of NK cells, potentially increasing susceptibility to PAD. Additionally, Fractalkine, also known as CX3C Chemokine Ligand 1 (CX3CL1), is a chemokine involved in the antitumor activity of lymphocytes, especially NK cells, T cells, and dendritic cells. Fractalkine induces angiogenesis and osteoclastogenesis, whereas CX3CL1 promotes the progressive infiltration of inflammatory cells into the artery wall (26, 27). Fractalkine may potentially reduce the risk of PAD. These findings align with existing research, where similar inflammatory profiles have been linked to other vascular diseases, thus supporting our conclusions. This alignment not only validates our results but also suggests these proteins could be universal targets across related diseases.

This research revealed several factors associated with PAD, including CC motif chemokine 19, CD5 T-cell surface glycoprotein, CUB domain-containing protein 1, Signaling lymphocytic activation molecule, Fibroblast growth factor 23, Interferon gamma, Interleukin-15 receptor subunit alpha, Interleukin-17C, Interleukin-1-alpha, Interleukin-5, Latency-associated peptide transforming growth factor beta 1, Matrix metalloproteinase-10, among others.

Additionally, our findings highlight the role of CXCL9, which inhibits collagen accumulation in human pulmonary artery smooth muscle cells through its interaction with the chemokine receptor CXCR3 (28), suggesting a mechanistic pathway that may influence PAD development. To date, no changes in CC motif chemokine 19 expression have been reported in the context of PAD. A study by Xiong et al., which included 49 coronary artery disease (CAD) patients and 17 CAD-free subjects, found a negative association between soluble lymphocyte activation gene 3 (sLAG3) levels and CAD occurrence, but no correlation with disease severity (29).. Additionally, elevated levels of Fibroblast Growth Factor 23 (FGF23) are linked to an increased risk of PAD, with a hazard ratio of 2.26 for individuals in the highest quartile of FGF23 levels compared to the lowest (30). This association persists independently of other variables, as shown by strong correlations between serum FGF23 levels and atherosclerotic disease in the lower extremities of Chinese individuals with type 2 diabetes (31). Our study advances the field by using bidirectional MR to explore the causal relationships between various inflammatory proteins and PAD, highlighting areas where literature is still developing. Given the roles of these proteins, targeted therapeutic interventions could be developed to modulate their activity. Specifically, inhibitors for pro-inflammatory proteins and enhancers for protective proteins could provide new treatment pathways for PAD. Additionally, measuring these proteins could refine the assessment of PAD risk and personalize preventive strategies.

In conventional observational research, a notable challenge is the susceptibility to biases arising from confounding variables, as well as the possibility of reverse causation. Secondly, the dataset used in this research is the most extensive GWAS conducted thus far, encompassing diverse populations. This extensive dataset enables our findings to be applicable to a variety of populations. Moreover, the epidemiological impact of MR analysis is significant, and its usage is projected to increase in the future. Due to the increasing availability of genetic data and advancements in methodology, MR analysis remains an indispensable tool in elucidating causal links between risk factors and disease outcomes, thereby advancing our understanding of disease pathogenesis.

This study utilized bidirectional MR analysis, which effectively determined the direction of causal networks (32). Nevertheless, it is crucial to acknowledge certain limitations within this research. Initially, the use of abstract figures instead of original data hinders the ability to conduct subset analyses, such as distinguishing among different forms of PAD. Furthermore, there is a lack of basic demographic information and clinical presentation data, hindering further exploration into the causal relationship between circulating inflammatory proteins and PAD at a more granular level. Setting the FDR significance level at 0.2 is indeed higher than the traditional threshold of 0.05, which may increase the risk of false positives. This is a relatively lenient standard aimed at capturing as many potentially important regulatory relationships as possible while maintaining a certain level of false positive rate control. Consequently, we have taken a cautious approach in interpreting the results, emphasizing that these findings require further validation and replication experiments to confirm their reliability. At the same time, we also recognize the potential subjectivity and limitations of this choice and encourage readers to consider these factors when evaluating our results. These limitations could affect the generalizability of results and potentially compromise the precision of the study. Finally, despite a thorough literature review and the identification of numerous variables, there is a possibility of undisclosed variables that may have influenced the outcomes. Therefore, careful interpretation of the discoveries is essential. Further research is necessary to confirm the causal roles of these proteins in PAD and to explore the clinical efficacy of targeting these markers. Longitudinal studies and clinical trials focusing on these inflammatory proteins are crucial to move from association to causation and to develop effective interventions.

## Conclusion

Our study utilized bidirectional MR analysis to explore the causal relationships between inflammatory proteins and PAD. This approach effectively mitigated concerns about reverse causality and minimized confounding factors, enhancing the precision of our causal inferences. The findings provide crucial insights into PAD treatment and contribute to our understanding of its pathophysiology and underlying biological mechanisms.

## Data Availability

The original contributions presented in the study are included in the article/**Supplementary Material**. Further inquiries can be directed to the corresponding author.

## Supplementary material

Supplementary Figure 1 Scatter plots of inflammatory proteins on Peripheral Artery.

Supplementary Figure 2 Funnel plots of inflammatory proteins on Peripheral Artery.

Supplementary Figure 3 Leave-one-out sensitivity analysis of inflammatory proteins on Peripheral Artery Disease.

Supplementary Figure 4 Scatter plots of Peripheral Artery Disease on inflammatory proteins.

Supplementary Figure 5 Funnel plots of Peripheral Artery Disease on inflammatory proteins.

Supplementary Figure 6 Leave-one-out sensitivity analysis of Peripheral Artery Disease on inflammatory proteins.

Supplementary Table 1 Information regarding the MR investigation of PAD inflammatory proteins.

Supplementary Table 2 MR examination presents all positive outcomes along with details about the genetic variants utilized.

Supplementary Table 3 Detailed information on the SNPs used in the positive results of reverse MR analysis.

Supplementary Table 4 MR analysis results of 91 inflammatory proteins on PAD from GWAS.

Supplementary Table 5 MR analysis results of 91 inflammatory proteins on PAD from UK Biobank.

Supplementary Table 6 MR analysis results of PAD on 91 inflammatory proteins.
