# Supplementary material for: Causal association between circulating inflammatory proteins and peripheral artery disease: a bidirectional two-sample Mendelian randomization study
Source: Front Immunol. 2024 Aug 16;15:1432041. doi: 10.3389/fimmu.2024.1432041 (PMC11361930; doi:10.3389/fimmu.2024.1432041)

Supplementary Figure 3. Leave-one-out sensitivity analysis of inflammatory proteins on Peripheral Artery Disease (A) Fractalkine. (B) Natural killer cell receptor 2B4.

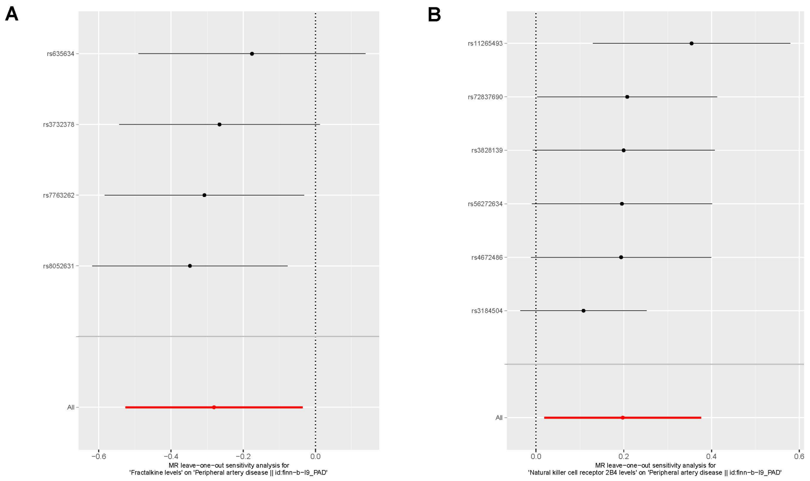

Supplement: Supplementary Figure 3 — Leave-one-out sensitivity analysis of inflammatory proteins on Peripheral Artery Disease. [file DataSheet3.pdf]
