# Supplementary material for: Causal association between circulating inflammatory proteins and peripheral artery disease: a bidirectional two-sample Mendelian randomization study
Source: Front Immunol. 2024 Aug 16;15:1432041. doi: 10.3389/fimmu.2024.1432041 (PMC11361930; doi:10.3389/fimmu.2024.1432041)

Supplementary Figure 5. Funnel plots of Peripheral Artery Disease on inflammatory proteins (A) C-C motif chemokine 19. (B) T-cell surface glycoprotein CD5. (C) CUB domain-containing protein 1. (D) Fibroblast growth factor 23. (E) Interferon gamma. (F) Interleukin-15 receptor subunit alpha. (G) Interleukin-17C. (H) Interleukin-1-alpha. (I), Interleukin-5. (J)Latency-associated peptide transforming growth factor beta 1. (K) Matrix metalloproteinase-10. (L) Signaling lymphocytic activation molecule.

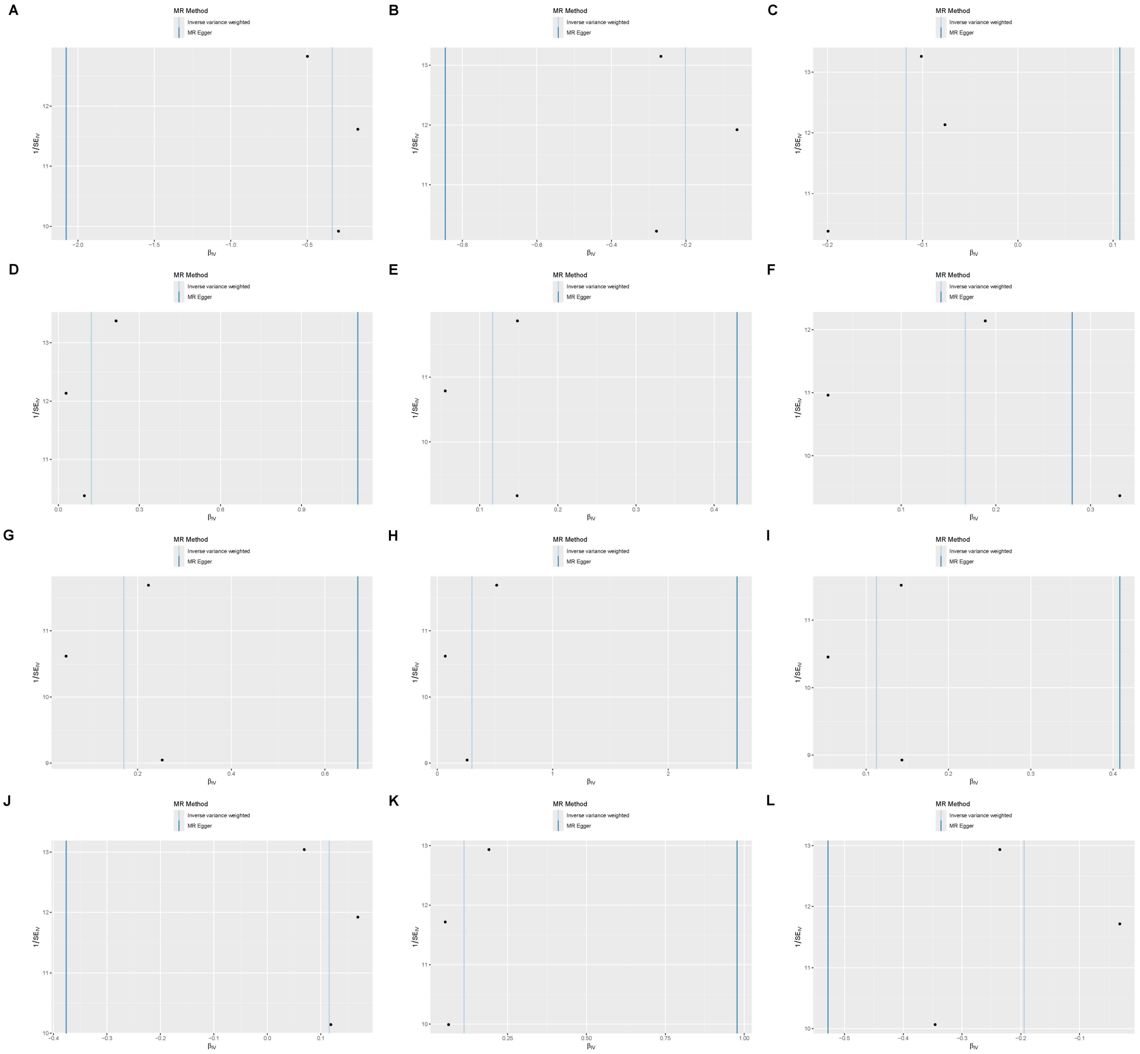

Supplement: Supplementary Figure 5 — Funnel plots of Peripheral Artery Disease on inflammatory proteins. [file DataSheet5.pdf]
